# Genome dynamics of multidrug-resistant *Acinetobacter baumannii* during infection and treatment

**DOI:** 10.1186/s13073-016-0279-y

**Published:** 2016-03-03

**Authors:** Meredith S. Wright, Alina Iovleva, Michael R. Jacobs, Robert A. Bonomo, Mark D. Adams

**Affiliations:** The J. Craig Venter Institute, La Jolla, CA USA; Department of Medicine, University Hospitals Case Medical Center, Cleveland, OH USA; Department of Pathology, University Hospitals Case Medical Center, Cleveland, OH USA; Department of Pathology, Case Western Reserve University, Cleveland, OH USA; Departments of Pharmacology, Molecular Biology and Microbiology, and the Center for Proteomics, Case Western Reserve University, Cleveland, OH USA; Louis Stokes Cleveland Department of Veterans Affairs Medical Center, Cleveland, OH USA

## Abstract

**Background:**

Limited treatment options are available for patients infected with multidrug (MDR)- or pan-drug (PDR)-resistant bacterial pathogens, resulting in infections that can persist for weeks or months. In order to better understand transmission and evolutionary dynamics of MDR *Acinetobacter baumannii* (Ab) during long-term infection, we analyzed genomes from a series of isolates from individual patients at isolate-specific, patient-specific, and population levels.

**Methods:**

Whole genome analysis of longitudinal isolates (range 2-10 isolates per patient spanning 0-829 days) from 40 patients included detection of single-nucleotide variants (SNVs), insertion sequence (IS) mapping, and gene content changes.

**Results:**

Phylogenetic analysis revealed that a significant fraction of apparently persistent infections are in fact due to re-infection with new strains. SNVs primarily resulted in protein coding changes, and IS events primarily interrupted genes or were in an orientation such that the adjacent gene would be over-expressed. Mutations acquired during infection were over-represented in transcriptional regulators, notably *pmrAB* and *adeRS*, which can mediate resistance to the last line therapies colistin and tigecycline, respectively, as well as transporters, surface structures, and iron acquisition genes.

**Conclusions:**

Most SNVs and IS events were isolate-specific indicating these mutations did not become fixed on the time scale investigated, yet over-representation of independent mutations in some genes or functional categories suggests that they are under selective pressure. Genome analysis at the population-level suggests that gene transfer including recombination also contributes to Ab evolutionary dynamics. These findings provide important insight into the transmission dynamics of Ab and the identification of patients with repeat infections has implications for infection control programs targeted to this pathogen.

**Electronic supplementary material:**

The online version of this article (doi:10.1186/s13073-016-0279-y) contains supplementary material, which is available to authorized users.

## Background

Bacterial pathogens resistant to multiple classes of antibiotics are increasingly detected in clinical settings, and patients with multidrug-resistant (MDR) infections have limited treatment options. This occurrence can translate into prolonged infections with increased morbidity and mortality [1, 2]. Analysis of MDR strains isolated over time from individual patients during infection and treatment can reveal important adaptations to the host environment [3–5]. The most extensive work to date has characterized longitudinal genome changes in cystic fibrosis patients infected with *Pseudomonas aeruginosa* and *Burkholderia* species, where these studies found that mutations were enriched in genes related to host adaptation and antibiotic resistance, and observed the co-existence of multiple lineages in the same host [6–10]. Patients with cystic fibrosis experience long-term infections, often spanning many years. Fewer studies have examined genomic changes in the context of acute infections that persevere during hospitalization, nor have longitudinal genomic changes within the context of a broader population structure been assessed.

In the last decade, *Acinetobacter baumannii* (Ab), one of the ESKAPE pathogens, emerged to become a predominant cause of nosocomial infections [11–14]. This is in part due to a remarkable rise in the frequency of MDR (resistant to at least three classes of antibiotic) and extreme drug resistant (XDR; resistant to all antibiotics except tigecycline or colistin) infections [12–14]. Notably, infections with carbapenem-resistant MDR and XDR strains are associated with longer hospitalization, greater economic costs, and higher mortality vs. carbapenem-susceptible strains [15–23].

Increased antibiotic resistance in Ab has been primarily driven by acquisition of resistance determinants encoded on mobile genetic elements and activation of intrinsic resistance mechanisms such as the chromosomal β-lactamases, *bla*_OXA-51-like_ and *bla*_ADC_, and increased activity of efflux pumps such as *adeABC* [24, 25]. A large ‘resistance island’ encoding more than a dozen antibiotic resistance genes was found in one of the first MDR Ab strains to be sequenced [26], and sequencing of hundreds of additional strains has shown that resistance genes are often associated with plasmids [27, 28]. The IS*Aba*1 element and other insertion sequences have also contributed to antibiotic resistance by activating expression of intrinsic resistance genes adjacent to their insertion sites [29]. IS*Aba*1 elements are abundant in some Ab strains, and the location of IS*Aba*1 insertions can add resolution to population dynamics and genomic epidemiology [30], but the role of these elements in adaptation to selective pressures during infections has not been addressed.

Clinicians have observed that in treating Ab infections, some patients remain chronically infected for prolonged periods of time. Whether long-term infected patients are the result of the persistence of one strain due to treatment failure, or new infection by a different strain, remains a pressing question. Genome sequencing provides an opportunity to address this question. We previously characterized the genetic profiles of Ab strains isolated from a large hospital system in the Midwestern USA and found considerable diversity including evidence for multiple closely related lineages and extensive variability of mobile elements [30]. In this follow-up study, we sought to understand what genome changes occur during the course of infection and antibiotic treatment. We therefore decided to sequence all available strains from patients that had multiple positive Ab cultures to characterize the nature of genetic change during infections. Longitudinal analysis of isolates collected during the course of infection and treatment can provide insight into infection history, and how Ab responds to host and antibiotic selective pressures.

## Methods

### Strains

*A. baumannii* strains identified in the University Hospitals Health System (UHHS) Clinical Microbiology Laboratory were stored in an archive since 2007. We identified all patients for which there were more than two isolates available in the archive as of 1 December 2013, and those isolates were selected for whole genome sequencing. A total of 136 new strains were sequenced and these genomes were combined for analysis with previously described sequences from UHHS [30] for a high resolution population-level analysis. Repeat cultures were ordered when patients presented with continued signs of infections. Clinical data, including treatment history and outcome, were collected from patient charts. The research study was reviewed and approved by the Institutional Review Board of the University Hospitals Case Medical Center, and conformed to the Helsinki Declaration.

### Sequencing

Overnight cultures were grown in LB broth and DNA was isolated using an UltraClean® Microbial DNA Isolation Kit (MoBio, Inc.) using a protocol implemented on an Eppendorf EpMotion automated pipetting device. Libraries were prepared for sequencing using Illumina NexteraXT kits and sequenced on an Illumina HiSeq 2000 with paired 100-base sequence reads. In general, more than 100-fold coverage was obtained for each genome. Each read set was assembled individually using *velvet* [31] and annotated using NCBI’s PGAAP pipeline (http://www.ncbi.nlm.nih.gov/genome/annotation_prok/). All genomes are available at NCBI under BioProject PRJNA262565.

### Phylogenetic analysis

A core phylogeny based on single nucleotide variants (SNVs) was inferred using SNVs identified by *kSNP* [32] and constructed using *RAxML* [33] with previously sequenced UHHS and other publicly available genomes included for phylogenetic context [30]. Genome positions with allele calls in at least 80 % of strains were included in the analysis. Regions of the genome subject to recombination were excluded from consideration in construction of the core phylogeny as in Wright et al*.* [30].

### Variant detection analysis

A more stringent approach was used to identify patient-restricted and isolate-specific SNVs from all patients with monophyletic isolates. Primary Illumina sequence reads were aligned to patient-specific reference genome assemblies, typically the earliest isolate from each patient, using *bwa*. To avoid the impact of low-quality sequences at contig edges, SNVs within 200 bases of contig edges were excluded. Variant-calling was performed using *freebayes* (arXiv:1207.3907 [q-bio.GN]) with a requirement for ≥20-fold coverage of the alternative allele with no reads supporting the reference allele, and a minimum SNV quality of 500. SNVs identified when reads from the reference genome were aligned to the reference contigs were excluded as likely indicating errors in the reference assembly. Patient-specific SNVs were identified as those SNVs uniquely shared within a patient based on the kSNP output for the full set of SNVs annotated to the reference Ab ACICU genome. ACICU was the first sequenced MLST ST2 (Global Clone 2) isolate, obtained from Italy in 2005 [34], and was selected as a reference because the majority of isolates in this study are GC2. Synonymous/non-synonymous status was inferred based on alignment of flanking sequences to the ACICU annotation.

### Genome variation analysis

To identify gene gain and loss events during infection, pan-genome analysis was performed using PanOCT [35]. The locations of IS elements in the draft genome assemblies was performed using ISSeeker, a custom perl script (https://github.com/JCVI-VIRIFX/ISseeker). IS locations in each genome were mapped to the common reference genome ACICU. The two most abundant IS elements in this population, IS*Aba*1 and IS*26* were examined and isolate-specific and patient-specific insertion sites were determined and mapped to the ACICU annotation.

### Functional enrichment analysis

Functional roles and pathways were assigned using KEGG designations [36] and TIGRfams [37] for each non-synonymous SNV and non-neutral IS event, and the number of genes within each category was determined for ACICU. To identify statistically significant enrichment in functional classes of genes, hypergeometric probability tests were conducted. In the calculation, the population size was the number of all genes in the PanOCT shared clusters for the 136 genomes (553,343 genes); the population successes value was calculated as the number of genes in each functional role or pathway category based on its abundance in ACICU multiplied by each genes’ occurrence in the PanOCT shared clusters; sample size was the number of SNV or IS events for isolate-specific (IS), patient-restricted (PR), or patient-specific (PS) categories, respectively; and the number of sample successes was the number of hit genes in each category.

## Results

### Isolate characteristics

**Fig. 1:**
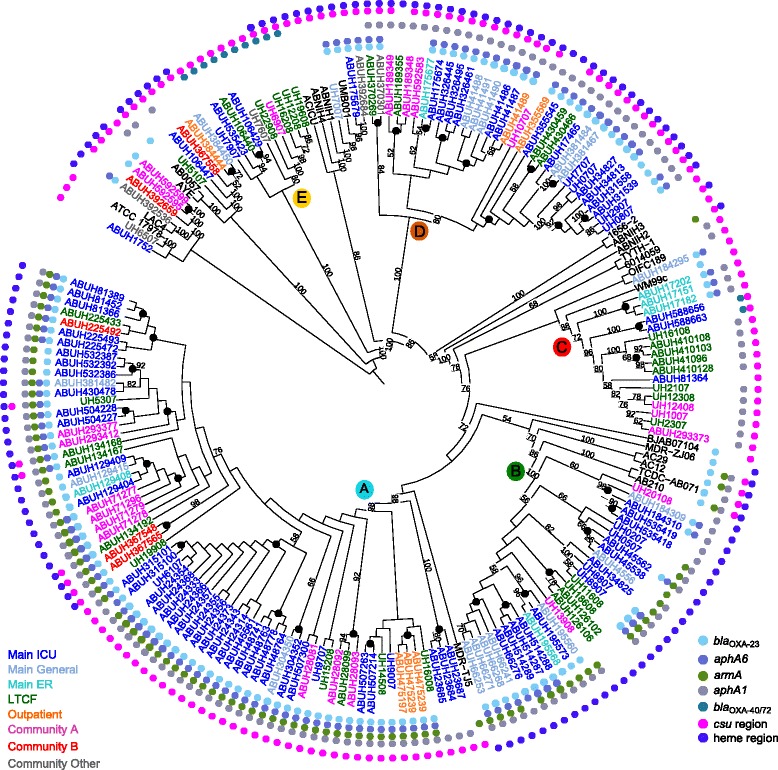
Core SNV phylogeny of *A. baumannii* strains. A core phylogeny was constructed using 50,472 SNVs that were present in at least 80 % of all strains. SNVs were identified using *kSNP* and the tree was built using RAxML. Clade A-E designation reflect primary clade structure previously designated [30, 44] where strains in Clade A-D through the ACICU branch are all Global Clone II, or multi-locus sequence type (MLST) ST2, strains [60]. Internal nodes with bootstrap support of at least 50 % are indicated. Black circles near terminal nodes indicate highly-supported monophyletic groups of strains from individual patients. Strain names are color-coded based on the hospital from which the isolate was obtained and the first the numbers after ABUH refer to patient and strain identifiers in Additional file 1: Table S2. Colored dots represent the presence of specific genes or genomic regions of interest as listed in the key

Genome sequences were obtained for 136 Ab isolates from 40 patients (Additional file 1: Table S1 and Additional file 2: Table S2). The number of isolates per patient was in the range of 2-10, over 0-829 days between first and last isolation (Additional file 1: Table S1). The pattern of antimicrobial susceptibility indicated that 125/136 isolates were resistant to ≥3 classes of antibiotics and are thus considered MDR. Isolates were collected from an integrated healthcare system: a central referral hospital (92/136), outlying community hospitals that were part of the healthcare system (23/136), long-term care facilities (16/136), and outpatient clinics (5/136). The isolates were collected from patients evaluated in an ICU, general medical wards, and from the emergency department. The 136 genomes were placed in the context of other UHHS genomes detailed in Wright et al. [30], and 22 publicly available reference genomes using a phylogeny constructed from 50,466 core SNVs located in non-recombinant regions of the genome (Fig. 1).

**Fig. 2:**
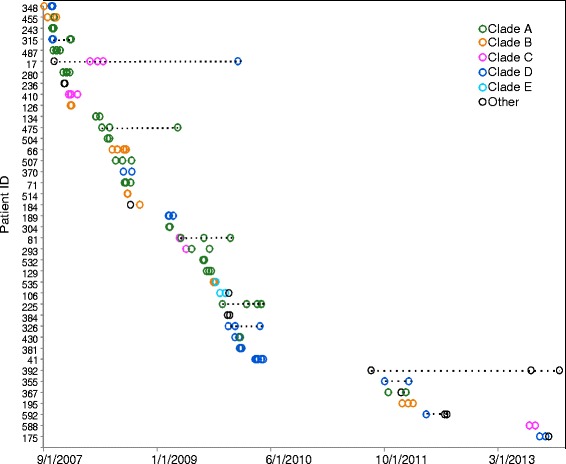
Date of isolation for each longitudinal isolates for each patient. Clade designation was determined as described in Fig. 1

A patient was considered to have an infection stemming from one colonization event when all of the isolates from that patient were located on a monophyletic, well-supported terminal node of the core phylogeny. Twenty-five patients had such monophyletic infections (Fig. 2, Additional file 1: Table S1). Fifteen patients had polyphyletic infections with isolates located at clearly distinct branches of the phylogenetic tree, indicative of at least two independent colonization events. Two of these patients have co-occurring polyphyletic infections in which phylogenetically distinct strains were isolated in overlapping time intervals, while the other 13 polyphyletic patients were likely re-infected with new strain types. In some cases, multiple related (monophyletic) strains were isolated from patients that were also infected with genetically distinct strains (polyphyletic). There was no relationship between the number of days separating culture-positive isolations and the likelihood of whether a new isolate was closely related (monophyletic) or represented a reinfection (polyphyletic) (Additional file 1: Table S1). The site of infection also did not predict whether subsequent isolates would be related: there are examples of both monophyletic (for example, Patient 236) and polyphyletic infections (for example, Patient 348) occurring at different body sites in the same patient.

### Monophyletic isolates

**Fig. 3:**
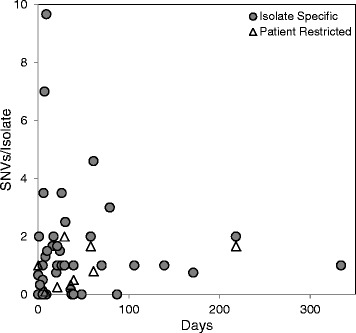
Relationship between SNV frequency and time between strain isolations The number of within-patient SNVs is shown as a function of time for isolate-specific and patient-restricted SNVs for each patient

SNVs in monophyletic groups from individual patients were of particular interest and were examined in more detail. Three classes of SNVs were defined: isolate-specific (IS) SNVs were found only in a single isolate; patient-restricted (PR) SNVs were found in multiple isolates (but not all) from a single patient; and patient-specific (PS) SNVs were shared by all isolates from a patient. For all three classes, the SNVs were not observed in any other UHHS or public reference strain. The complete list of these SNVs with gene annotations is presented in Additional file 3: Table S3. Most of the SNVs that occurred during infection were unique to each isolate: of the 220 detected SNVs, 189 (86 %) were isolate-specific. IS SNVs arising during infection were detected in 31 strains (mean 4.8 SNVs, range 0-29 SNVs/patient). PR SNVs were identified in nine patients (range: 0-8 SNVs/patient). There was no relationship between the number of SNVs per isolate and the number of days between isolations (Fig. 3). For example, the three isolates from Patient 236 collected on the same day had eight SNVs among them, while three isolates from Patient 195 over 48 days had 0 detected SNVs among them. Isolates from distinct body sites did have more isolate-specific SNVs. For example, the two Clade B isolates from Patient 184 had 14 (percutaneous endoscopic gastrostomy site) and 15 (urinary tract infection) IS SNVs. SNVs that were restricted to a patient had a higher proportion of non-synonymous (NS) amino acid substitutions compared to synonymous (S) substitutions (NS/(NS + S): 0.97) than the isolate-specific (0.77) and patient-specific SNVs (0.74) (Table 1) yet all categories of isolate- and patient-restricted SNVs were strongly skewed towards non-synonymous mutations relative to the population-level NS/(NS + S) ratio of 0.18.

**Table 1:** SNV and IS event summary

SNV	Intergenic^a^	Synonymous	Non-synonymous	NS/(N + S)
Isolate-specific	30 (16)	36	123	0.77
Patient-restricted	2 (7)	1	28	0.97
Patient-specific	58 (15)	83	237	0.74
Population-level^b^	10,709 (9)	85,865	19,190	0.18
IS*Aba*1^c^	Overexpressed	Loss of function	Neutral	Total
Isolate-specific	8 (33)	13 (54)	3 (13)	24
Patient-restricted	2 (67)	0	1 (33)	3
Patient specific	8 (32)	10 (40)	7 (28)	25
Population-level	13 (15)	50 (60)	21 (25)	84

^a^Values in parentheses are the percentage of SNVs that are intergenic

^b^Summary from kSNP output analyzing the 136 longitudinal isolates, and UHHS and public genomes

^c^Values in parentheses are the percentage of IS events in each category

IS*Aba*1 is abundant and dynamic in the UHHS population, and mapping of the insertion sites for IS*Aba*1 revealed additional genomic modifications during infection. Similar to SNV patterns, most new IS*Aba*1 insertions were isolate-specific and these tended to be in coding regions or in an orientation that would result in overexpression of an adjacent gene (Table 1, Additional file 4: Table S4). Only three patient-restricted IS events were detected, two of which are predicted to result in the overexpression of downstream genes. Independent IS*Aba*1 insertions upstream of the *fimA* gene in two different patients were predicted to lead to overexpression of the type-1 fimbrial protein. A smaller fraction of patient-specific IS events (uniquely shared among all isolates from a patient) were in coding sequences or an overexpression orientation, while across all UHHS isolates, IS*Aba*1 was less likely to be found in an overexpression orientation.

The 10 strains isolated from Patient 243 provide particularly illustrative examples of SNV and IS*Aba*1 patterns associated with temporal and spatial heterogeneity. Six isolates encompassing respiratory, urine, and abdominal sites of infection had no detectible SNVs among them, including three isolates cultured 8 days after the initial sample. The other four isolates had between two and six SNVs relative to Day 0 isolates. All but one these SNVs were isolate-specific, where the mutation in the RNA polymerase gene *rpoB* was present in two Day 8 abdominal fluid isolates. No IS*Aba*1 insertions that arose during infection were shared among Patient 243 isolates. SNV patterns from Patient 236, on the other hand, reveal that spatial isolation can lead to strain differentiation. The three isolates are monophyletic, forming a terminal branch with distinct SNVs and gene content from other UHHS strains. The blood and abdominal fluid isolates (ABUH23684 and ABUH23685) have three shared SNVs and one IS*Aba*1 insertion not present in ABUH23687 (a scrotal isolate) which has two unique SNVs.

Gene content changes in monophyletic isolates were primarily gene loss events centered around IS*26*-mediated deletions in the *astA* and ACICU_02399 Tn*1548* resistance island regions described in [30]. IS*26* has been shown to cause deletions of adjacent sequence [38, 39]. We observed a partial deletion of Tn*1548* at the *catB* gene in ABUH315100 which resulted in the loss of the amikacin resistance gene, *armA*, and independent losses of the adjacent *csuE* region in multiple isolates as best depicted by the variable presence of *csuE* in isolates from clades B and D (Fig. 1). Variation in phage content was observed in Patient 410 in a region previously described as dynamic [30]. The first Patient 410 isolate had one 48 kbp region of phage sequence, the second had an additional 36 kbp of sequence, and the last two isolates had an additional 13 kbp of sequence in this region, resulting in a variable phage region in the range of 48-97 kbp in size.

### Polyphyletic isolates

Two patients had co-occurring polyphyletic infections in the same infection site: bronchial specimens from Patient 367 and Patient 455 produced isolates from different clades. There was no evidence for genetic exchange between the Clade A strains in Patient 367 and the more divergent ABUH367558, which was isolated at an intermediate time point between the two clade A strains. The two clade A strains are isogenic, as they share a terminal branch on the tree, with only three SNVs that distinguish them and a uniquely shared IS*Aba*1 insertion in a gene encoding the histidine kinase protein of an uncharacterized two component regulatory system (TCRS) (ACICU_03157). This strain persisted largely unchanged during the 77 days between isolations. Genetic exchange did occur between the two different strain types in Patient 455: pABUH1 was transferred from the clade A strain to the clade B strain (ABUH45561 to ABUH45538/ABUH45562) as previously described [30].

Thirteen patients had polyphyletic infections where re-infection or replacement with a new strain type was likely. Clade A strains carry the most resistance determinants and were not replaced with a different strain type in this population.

### Enrichment and convergence

**Fig. 4:**
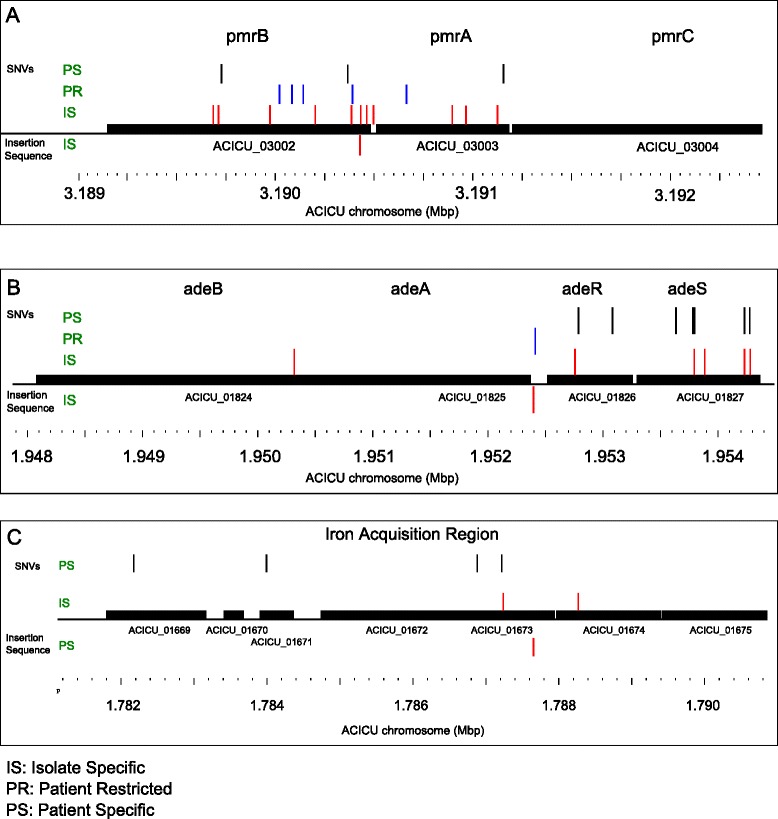
Selected loci with multiple independent mutations. Genes and gene regions that exhibited a significantly over-representation of mutation frequency are shown. **a** The *pmrCAB* genes encode the PmrAB TCRS that is associated with resistance to colistin and one of the target genes (*pmrC*), that encodes a lipid A phosphoethanolamine transferase. **b** The *adeABC* genes encode an RND family efflux pump that is involved in resistance to several classes of antibiotics. The *adeRS* genes encode a TCRS that regulates *adeABC*. **c** A region encoding several genes involved in siderophore biosynthesis. Iron acquisition mediated by siderophores is an important adaptive mechanism in the host environment that is characterized by limited free iron concentrations

Analysis of the functional categories of genes with SNVs and IS insertions demonstrated that genes associated with antibiotic resistance and host interaction are over-represented (Table 2). The most commonly mutated genes were *pmrAB* that encode the two component regulatory system (TCRS) involved in mediating colistin resistance through modification of surface polysaccharides and membrane charge [40–43]. Twelve patients had at least one isolate with a mutation in *pmrA* or *pmrB* and four of those patients had multiple strains with independent mutations (Fig. 4a). The *adeRS* genes, which encode a TCRS that regulates expression of the efflux pump operon *adeABC*, were the second most commonly mutated loci, found in isolates from 12 patients (Fig. 4b). Seven patients had mutations in both the *pmrAB* and *adeRS* TCRS genes. Another uncharacterized histidine kinase component of a TCRS, ACICU_03157, had both IS*Aba*1 and point mutations in three patients. Transporters were also significantly enriched, accounting for 30 out of 123 non-synonymous SNVs in the isolate-specific SNV set. This included multiple cation (*copA*, *znuB* (two independent mutations) and *arsB*) and iron-related transporters (*fhuE* (two independent mutations), *feoB*, and siderophore transporters), as wells as antibiotic transporters including the porin *carO, adeB*, and *adeJ* efflux pumps, and those associated with lipopolysaccharide export, *msbA* and *lptF*. The iron acquisition gene, *iucD*, had both NS SNV and IS mutations in strains from four patients (Fig. 3c). Motility and adhesion related genes were also significantly enriched (for example, *csuE*, *pilG*, and *fimA*). Mutations in the tyrosine kinase *wcz* gene involved in capsular polysaccharide assembly were detected in three patients, while UDP-N-acetylmuramate-alanine ligase *murD* mutations were observed twice. The RNA polymerase III gene, *rpoB*, had three NS mutations in two patients; all of the SNVs were located in the rifampin resistance-determining region (RRDR).

**Table 2:** Enrichment analysis of genes with SNV and IS events. Significant enrichment in classes and pathways assessed using hypergeometric distribution test based on abundance of genes in each category in ACICU, integrated with PanOCT shared clusters report (see text for detail)

		SNVs			IS*Aba*1		
	Number of genes in ACICU	Isolate-specific	Patient-restricted	Patient-specific	Isolate-specific	Patient-restricted	Patient-specific
Transporters	282	30***	4	23*	3	0	2
Transcription factors	207	5	2	20**	2	0	4*
Two component regulatory system	26	19***	5***	11***	2	0	4
Lipid A and capsular polysaccharide synthesis	54	7**	2*	5	1	0	0
Iron transport	64	8**	1	4	0	0	1
Motility	31	4*	1	3	1	1	1
Total non-synonymous and non-neutral events used in hypergeometric test calculations		123	28	215	21	2	18

* <0.05, ** <0.005, *** <0.0005

IS*Aba*1 insertion sites also tended to be in genes with functions in antibiotic resistance and host adaptation. Genes encoding transporters (including AdeABC efflux pump), transcriptional regulators, surface-associated genes, and secreted and adhesion genes like *papD* and *fimA*, were all modified by IS*Aba*1 (Additional file 4: Table S4).

### New insights into the population structure of *A. baumannii* population at UH

The primary clade structure described previously is unchanged with the addition of the strains analyzed here [30, 44]. Clade A is most abundant, with 58 isolates. The analysis of the expanded set of UHHS strains revealed that there are two clade A subgroups that differ in the presence of a region that encodes a heme oxygenase, *hemO*, (Fig. 1) that was previously described as a recombination hotspot [30, 45, 46]. There is evidence for more divergent strains among the most recent isolates that carry new genetic material, including the *bla*_OXA-72_ variant of the *bla*_OXA24/40_-type carbapenemase gene in ABUH588656 and ABUH588663. There is now evidence for recombination at the *bla*_OXA-51_ locus in clade C, with a different allele now present.

Epidemiological and genomic data highlight multiple cases of likely patient-to-patient transmission. For example, the clade A cluster of isolates from Patients 243, 315, and 487 all occurred in the ICU within the span of a few days in 2007. These three patients share four unique SNVs, while Patients 243 and 487 share an IS*Aba*1 event. Similarly, Patient 66 and Patient 514 overlapped in the ICU in 2008 and had nearly identical clade B isolates with one shared SNV (1 bp upstream from start of ACICU_01396, a predicted MFS transporter).

Patients with persistent infections move among hospital locations during the course of treatment. Eleven patients contributed isolates obtained in more than one hospital, with transfer between a long-term care facility (LTCF) and the tertiary care hospital being the most common transfer route (four patients moved to LTCF, four patients were transferred from a LTCF to the main or regional hospitals). Patient 392 was infected with three different strains over the course of >800 days, each isolated during a stay at a different community hospital. Patient 225 was infected with a monophyletic set of strains from clade A over 170 days, with strains isolated during the patient’s stay at a LTCF, the main ICU ward, a community hospital, and again at the main ICU ward.

## Discussion

Two lines of evidence suggest that natural selection is contributing to the range of SNVs and IS insertions observed in the series of longitudinal isolates from each patient. First, genes involved in antibiotic resistance and host interaction were significantly enriched for novel genetic variants. MDR strains have limited treatment options, resulting in an increased reliance on last-line therapies like colistin and tigecylcine. The *pmrAB* genes were the most frequently mutated, with 18 independent mutations and IS events observed. These mutations tended to occur in patients that had been treated with colistin: 12 patients had isolates with mutations in these loci, and at least nine of them received colistin therapy. Colistin treatment status was unknown for the other three patients due to incomplete patient histories. Mutations in *pmrAB* that arise during treatment have been described previously [47]. Ample evidence supports a role for the TCRS system PmrAB in regulation of colistin resistance in Ab through upregulation of *pmrC*, a lipid A phosphoethanolamine transferase [41, 42]. Some relationships between *pmrAB* mutation and colistin minimum inhibitory concentration (MIC) were observed. For example, the MIC for isolate ABUH41489 with an IS*Aba*125 interruption of *pmrB* was >128 ug/mL, while isolate ABUH41488 from the same patient, which had a *pmrA* mutation (I173F) had a MIC measured at 2 ug/mL. However, the majority of strains with *pmr* mutations had MIC values <2 μg/mL, which is considered phenotypically susceptible in standard clinical assays (Additional file 2: Table S2). A recent study of the development of resistance in the context of colistin therapy in *A. baumannii* showed that clinical colistin resistance can go undetected by standard clinical methodologies [48]. Though phenotypically susceptible, *pmrC* expression values were significantly increased from 5-30-fold in three strains tested relative to their isogenic parental strains from the same patient (RNASeq data not shown). Thus the elevated *pmrC* expression suggests that *pmrAB* mutations in these strains may result in lipid A modifications that confer some level of protection from colistin. Mutations in *lpxACD*, encoding for lipid A biosynthesis pathway, have also been implicated in conferring colistin resistance [49]. We did not observe any mutations in the *lpx* genes, supporting the prediction that *pmrAB* mutations are likely to be the most commonly encountered colistin resistance mechanism in Ab in clinical settings due to a higher fitness cost of *lpxACD* mutations [50].

Another TCRS, *adeRS*, and its associated efflux system, *adeABC*, also had multiple independent mutations in this population, with 16 independent mutations and IS events in 12 patients. AdeRS regulates the expression of the *adeABC* efflux pump genes which are associated with resistance to several antibiotics including to tigecycline [51, 52]. Sequence variation is high in these genes as observed previously and related to tigecyline resistance [53]. Interestingly, only three patients with *adeRS* or *adeABC* mutations in this study were confirmed to have received tigecycline during treatment, while four did not, and five patients had inconclusive patient records. All *adeRS* and *adeABC* mutant isolates had tigecycline MIC values >2 μg/mL (Additional file 2: Table S2), indicative of a resistant phenotype. Eight of the 14 mutations were patient-specific, meaning that even the initial isolate had the mutation. This observation is consistent with the hypothesis that the regulation of *adeABC* is under selection for more than just tigecycline efflux [51]. Mutations in the *rpoB* gene encoding RNA polymerase III can confer resistance to rifampin. Three isolates from two patients had *rpoB* mutations in the rifampin resistance determining region (RRDR) [54] and both patients received rifampin therapy in combination with colistin. This suggests that mutations in other multiply-mutated genes may also have adaptive value in the context of infection.

MDR infections still have to persist in the context of the host immune system, and functional categories related to regulation of transcription, membrane transport, surface polysaccharide synthesis and organization, iron acquisition, and motility were enriched in mutations. Mutations were observed in the tyrosine kinase *wzc* gene involved in mediating capsular polysaccharide organization [55–57]. Notably, several mutations occurred in genes encoding iron acquisition and siderophores with potential implications for iron acquisition during host infection. The enrichment in transcription factors in SNV and IS events raises the question of how the transcriptional landscape changes during infection. For example, the histidine kinase component of an uncharacterized TCRS, ACICU_03157, had both SNVs and IS mediated changes in this population.

The second line of evidence of a role for natural selection in the set of observed SNVs is the elevated ratio of non-synonymous variants compared to the population-wide average (Table 2). Isolate-and patient-specific SNVs were much more likely to be non-synonymous than SNVs observed in isolates from multiple patients. SNVs in multiply-hit genes also have higher non-synonymous ratios at the population level compared to the population mean ((NS)/(NS + S) = 0.18) (Additional file 5: Table S5), suggesting that these genes may be subject to diversifying selection, in contrast to those genes below the population-wide value. For example the iron-related genes, ACICU_01673, ACICU_01696, ACICU_02572, and ACICU_02581, all have population-wide ((NS)/(NS + S) >0.35. Furthermore, siderophore synthesis genes, transporters, and TCRS, were also observed in strain-specific SNVs in an Ab outbreak [58]. Functional gene enrichment findings are also in agreement with other longitudinal studies including persistent and asymptomatic *Burkholderia pseudomallei* infection which had key changes in lipopolysaccharide biosynthesis and modification [10], and those observed during 12 weeks of *Staphylococcus aureus* blood stream infection that included transporters and antibiotic resistance genes [4]. Lieberman et al*.* (2014) found extensive diversity of *Burkholderia dolosa* in individual CF patients, with multiple adaptive mutations appearing, but few becoming fixed in the population over time [6]. In a longitudinal study of over 400 *Pseudomonas aeruginosa* strains isolated from 34 CF patients, convergent evolution was identified in several genes involved in host adaptation based on remodeling of regulatory networks [59]. An alternative explanation, however, for the elevated rate of non-synonymous mutations is that there has not been adequate time for deleterious mutations to be removed from the population. This alternative hypothesis predicts that over time the fraction of non-synonymous mutations would decline. More extensive sampling of infected patients will be required to better understand the spectrum of mutations that arise during infection and make inferences about their functional effect.

Hospital-acquired infections are generally considered acute and treatable. Increasing rates of antibiotic resistance are changing that perception. Strains resistant to all FDA-approved drugs now represent a significant fraction of Ab infections in many hospitals and have led to long-term infections that are difficult to treat. Relatively few studies have examined the evolution of bacterial pathogens during long-term infections. We found that patients persistently infected with MDR Ab are due to both treatment failure and re-infection with new strains. Multiple examples were found of patients infected with the same strain for >150 days. Patient movement data highlight the need to monitor patients with a history of MDR Ab as they are admitted to a new hospital unit.

A limitation of this study is that serial sampling of infected patients was not performed in a systematic or prospective manner, but relied on a convenience sample that may introduce biases. Another limitation is that a single isolate was sequenced from each culture date and so we were not able to evaluate the population of variants that were present in a patient at any given time. In some cases the retrospective sampling design does not distinguish between co-occurring, replacement, or re-infection scenarios. To more rigorously distinguish between these classifications, multiple isolates from multiple time points need to be analyzed. Nonetheless, we were able to show that both long-term infections and super-infections with genetically distinct strains are common.

## Conclusions

Genomic analysis of *A. baumannii* isolates collected from the same patient over time revealed enrichment in mutations associated with antibiotic and host responses, including those encoding for transcriptional regulators, cell surface structures, and iron utilization. Most mutations were isolate-specific, yet enrichment analysis suggests that the much of the genetic variation detected is under selection.

## Additional files

Additional file 1: Table S1. Summary of patient and isolate data. Clade designation and infection type (monoclonal, polyclonal, mixed) is based on strain relatedness in the core phylogeny (Fig. 1). Number of SNVs (isolate-specific, patient-restricted, patient-dependent); number of IS*Aba*1 events (isolate-specific, patient-restricted, patient-dependent). (XLSX 32 kb) Additional file 2: Table S2. Isolate metadata including date and location of isolation, and antibiotic susceptibility data. Colistin susceptibility was assayed using both E-test strips (bioMérieux) and broth microdilution tests (Sensititre platform). (XLSX 63 kb) Additional file 3: Table S3. Isolate-specific, patient-restricted, patient-specific SNVs. (XLSX 64 kb) Additional file 4: Table S4. Isolate-specific, patient-restricted, patient-specific IS*Aba*1 events. (XLSX 17 kb) Additional file 5: Table S5. Multiply mutated genes. All genes with multiple SNV and IS*Aba*1 events are listed with their predicted function. The text color indicates what the class of mutations are: SNV or IS*Aba*1 events, and whether the mutation was isolate-specific, patient-restricted, or patient-dependent. Green: IS SNV; blue: PR SNV; purple: PS SNV; orange: IS IS*Aba*1; brown: PR IS*Aba*1; pink: PS IS*Aba*1. (XLSX 20 kb)
